# Gut Microbial Genetic Variation Regulates Host Reproduction

**DOI:** 10.1111/1751-7915.70248

**Published:** 2025-10-10

**Authors:** Xiaoyue Ding, Yalun Wu, Dianshuang Zhou, Rongrong Gu, Tao Zhu, Wen Cai, Yuxuan Ren, Ying Li, Chuhe Wang, Anqi Tan, Ying Li, Zuobin Zhu

**Affiliations:** ^1^ Jiangsu Engineering Research Center for Precision Diagnosis and Treatment of Polygenic Critical Diseases, Key Laboratory of Genetic Foundation and Clinical Application, Department of Genetics Xuzhou Medical University Xuzhou China; ^2^ Medical Technology College Xuzhou Medical University Xuzhou China

**Keywords:** *C. elegans*, cyclophosphamide (CTX), genetic variation, gut microbiota, male reproduction, purine metabolism pathway

## Abstract

The gut‐testis axis enables gut microbes to influence host reproduction; nonetheless, the specific role of microbial genetic variation in this process remains elusive. In this study, using *Caenorhabditis elegans* ( *C. elegans* ) as a model organism, we identified 46 *Escherichia coli* ( *E. coli* ) strains that markedly enhanced *C. elegans* fertility. Of them, 26 strains were mutant variants capable of mitigating cyclophosphamide (CTX)‐induced reproductive disorders in *C. elegans* . To investigate their application, we constructed probiotics to validate their effectiveness in mouse reproduction. The engineering probiotic Ecn Δ*pal* significantly improved spermatogenesis in mice with CTX‐induced reproductive disorders. Finally, comprehensive metabolome and transcriptome analysis suggested that the purine metabolism pathway may contribute to ameliorating cyclophosphamide‐induced male reproductive toxicity. Overall, our study provides novel insights into the impact of gut microbial genetic variation on host reproduction and elucidates novel therapeutic avenues for mitigating CTX‐induced male reproductive toxicity.

## Graphical abstract

We discovered that microbial genetic variation interacts with host genetics to regulate host reproduction. We identified *E. coli* mutants that mitigate cyclophosphamide‐induced male reproductive toxicity and found that the purine metabolism pathway may contribute to it.

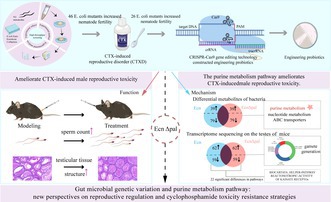

## 1 Introduction

Increasing evidence emphasises the impact of gut microbiota on male reproductive function through mechanisms, including hormone regulation, immune system conditions, nutrient metabolism and inflammation (Cai et al. 2022; Tanoue et al. 2019; Wang and Xie 2022). Additionally, gut microbiota has shown promise in directly improving host fertility. Modifying the gut microbiota in mice through faecal microbiota transplantation alters the systemic environment for sperm development, enhancing sperm quantity and quality (Hao et al. 2022). Additionally, feeding elderly male mice purified microorganisms, such as *Lactobacillus reuteri* , restored the testosterone level to that observed in younger mice (Poutahidis et al. 2014). However, the therapeutic potential of gut microbiota remains limited by challenges in standardising co‐probiotic transplantation techniques and the insufficient characterisation of specific probiotic strains (Madhusoodanan 2020; Nath et al. 2022).

Microbial genetic variations regulate the co‐evolutionary relationship between intestinal microbes and their hosts, enabling microbes to adapt to host environment changes, modify metabolic functions and fine‐tune host–microbe interactions (Groussin et al. 2020; Henry et al. 2021; Shapira 2016). These genetic variations can affect host reproduction by producing metabolites and regulating hormones. However, considering the complexity and slow mutation rates of microbial genes, identifying substantial genetic alterations within short timeframes remains challenging. Understanding of the mechanism by which microbial genetic variations affect host reproduction is limited, leaving a critical knowledge gap concerning the microbial factors that regulate host reproduction. Closing this gap is central to exploring the mechanisms underlying male infertility.

Gene mutations in *Escherichia coli* can substantially affect the development and lifespan of its host, *Caenorhabditis elegans* (nematode) (Han et al. 2018; Zhang et al. 2019). This strategy presents a relatively effective and precise method for functionally regulating the gut microbiota by manipulating specific genes within the gut microbiota. *C. elegans* is a nontoxic, harmless, hermaphroditic, rod‐shaped nematode that feeds on bacteria. It takes only 2.5 days to develop from a fertilised egg to an adult. After hatching, the larval stage is primarily divided into four stages, namely L1, L2, L3 and L4, followed by the adult stage (Boyd and Williams 2003; Lemieux and Ashrafi 2016; Marsh and May 2012). The simplicity of gut microbiota in *C. elegans* makes it an excellent model organism for investigating the relationship between microbial genetic variations and host reproduction. The *E. coli* Keio Knockout Collection consists of single‐gene knockout mutants of all nonessential genes in *E. coli K‐12 BW25113*, encompassing over 3000 genes (Baba et al. 2006). Each mutant strain produces diverse metabolites, forming an extensive library of material resources and affecting host life activities. These mutations can be genetically engineered into probiotics for clinical application. *E. coli* Nissle 1917 (Ecn), a well‐studied probiotic, was isolated in 1917 by German bacteriologist Alfred Nissle. He developed the live biotherapeutic product Mutaflorthat, comprising the *E. coli* strain Nissle 1917 as the active ingredient (Sonnenborn 2016).

Drug‐induced reproductive toxicity is a major cause of male infertility. Cyclophosphamide (CTX)—a cytotoxic alkylating agent—is among the most aggressive chemotherapeutic agents, extensively used as an antineoplastic drug for cancer treatment (Ludeman 1999). Despite its broad clinical applications, CTX may cause toxic adverse effects in multiple organ systems, including the testes, leading to male infertility (Madondo et al. 2016). Particularly, it affects men of reproductive age with cancer by jeopardising their ability to conceive offspring. Understanding the association between microbial genetic variation and host reproduction not only elucidates the impact of microbial genetic variation on host reproduction but also facilitates genetically engineered probiotic development to mitigate CTX‐induced male reproductive toxicity.

In this study, we developed a high‐throughput screening platform to identify *E. coli* mutants that regulate *C. elegans* reproduction. Additionally, we identified mutants that mitigate CTX‐induced male reproductive toxicity, with purine metabolism serving as a key pathway. Our findings elucidate promising probiotic candidate strains and treatment strategies for addressing male infertility and mitigating CTX‐induced male reproductive toxicity.

## 2 Results

### 2.1 High‐Throughput Screening Identified *E. coli* Mutants Regulating *C. elegans* Reproduction

**FIGURE 1:**
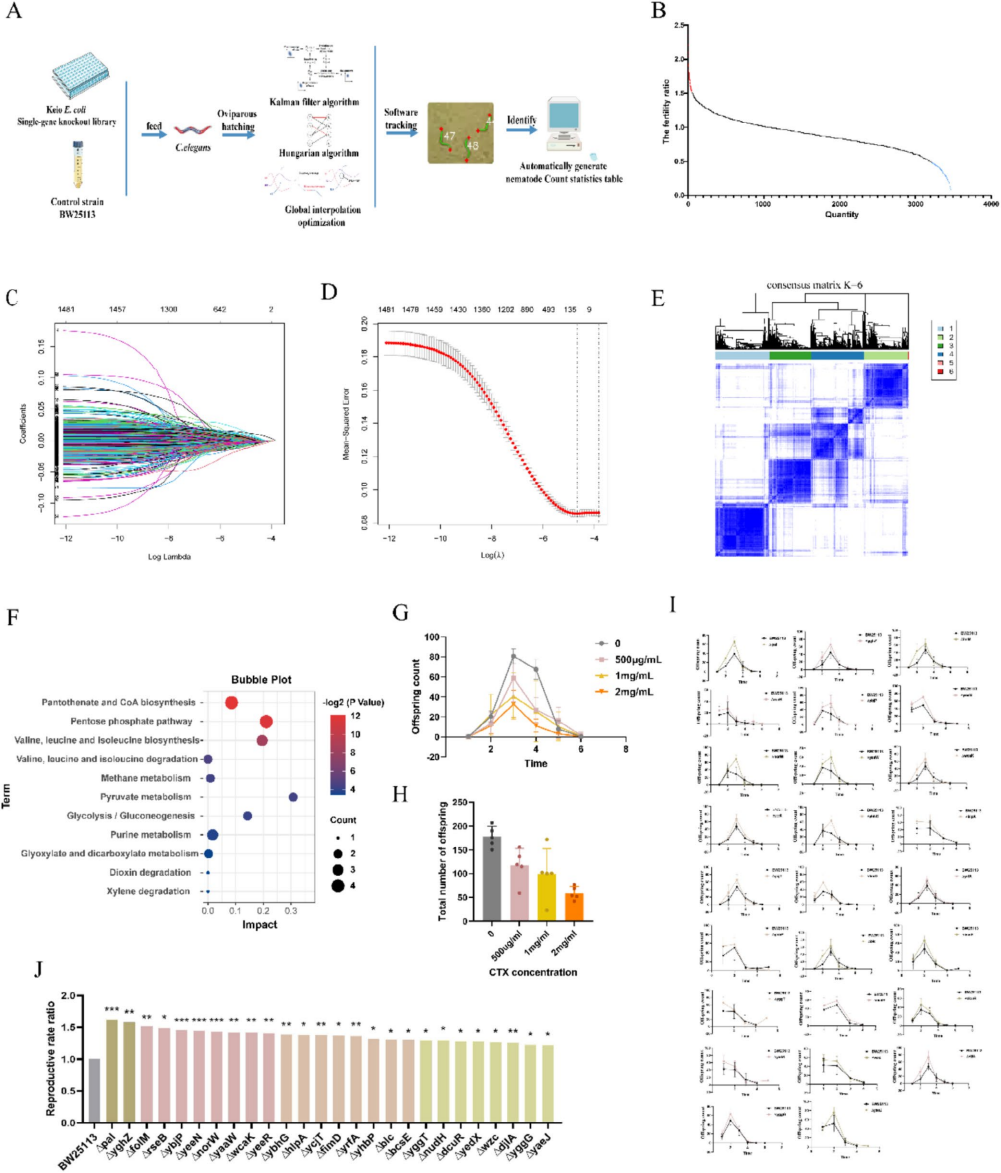
High‐throughput screening of *E. coli* mutants that regulate *C. elegans* fertility (A) Schematic flowchart of the high‐throughput screening for single‐gene knockout *E. coli* strains that improve fertility in N2 *C. elegans* . (B) Mutant *E. coli* strains significantly regulate the fertility of *C. elegans* from 3467 single‐gene knockout *E. coli* strains. The fertility ratio is calculated as the total number of nematode offspring after being fed on mutant bacteria divided by the total number of offspring after feeding on BW25113. The fertility ratio of 46 mutant strains exceeding 1.5 is highlighted in red and fertility below 0.5 of *E. coli* mutants is highlighted in blue. (C) Coefficient variability in LASSO regression. Each line represents a variable, with the corresponding number displayed at the end. The ordinate indicates the coefficient, whereas the upper abscissa indicates the number of nonzero coefficients under different regularisation parameters. The lower abscissa represents a normalised regularisation parameter. The graph illustrates the trajectories of variable coefficients under different regularisation parameters. The larger the regularisation, the lower the model complexity. (D) Optimal parameter λ selection in the LASSO regression model using cross‐validation. The lower x‐axis represents normalised regularisation parameters, whereas the upper x‐axis represents the number of nonzero coefficients. The y‐axis represents the likelihood bias. The left dotted line corresponds to the lowest point of the curve (lambda.min), providing the smallest average cross‐validation error. The right dotted line (lambda.lse) represents the value within one standard deviation of the minimum error. (E) Comparison of clustering results with real features. * represents test values of hypergeometric distribution (*p* < 0.05). (F) Bubble map of KEGG functional enrichment for 29 differential metabolites. The abscissa represents the enrichment rate, the ordinate represents the enrichment pathways, bubble colour (blue to red) indicates an increasing enrichment rate. The dot size indicates the number of genes in each pathway. (G) Total offspring number in N2 nematodes fed with different concentrations of CTX. (H) Daily offspring number in N2 nematodes fed with different concentrations CTX. CTX at 1 mg/mL reduces the total offspring number in N2 nematodes by approximately 50%. (I) Effect of 26 single‐gene knockout *E. coli* strains and control strains on the reproductive cycle of N2 nematodes. (J) The ratio of the total offspring number N2 nematodes fed with 26 selected *E. coli* single‐gene knockout strains to control strains. Strains increasing fertility are classified as follows: Yellow (55%), pink (40%), orange (30%) and green (20%). *n* = 10, using t‐test statistical analysis, * represents t‐test value *p* < 0.05, ** means *p* < 0.01 and *** means *p* < 0.001. LASSO, Least Absolute Shrinkage and Selection Operator; KEGG, Kyoto Encyclopedia of Genes and Genomes CTX, cyclophosphamide; CTXD, CTX‐induced reproductive disorder.

Using the *E. coli–C. elegans* high‐throughput screening platform, single‐gene knockout mutant strains from the *E. coli* Keio Knockout Collection, along with the parental strain BW25113, were fed to N2 nematodes. Worm Studio—a nematode counting software developed by our research group—recorded the total number of offspring produced by nematodes on day 4. At this point, parent nematodes stop laying eggs, and their offspring have not yet started laying eggs (Figure 1A). The total number of offspring produced by nematodes (fed with mutant strains) exceeded 50% of the control group (fed with BW25113). The fertility ratio was defined as the ratio of the total number of offspring produced by N2 nematodes fed with mutant strains to that produced by N2 nematodes fed with control strains. Mutant strains were categorised as those increasing nematode fertility when the ratio exceeded 1.5. Conversely, strains with a fertility ratio below 0.5 were categorised as those reducing nematode fertility. Among the 3467 tested strains, primary screening identified 46 *E. coli* mutants (highlighted in red) that increased nematode fertility and 59 mutants (highlighted in blue) that reduced fertility (Figure 1B).

To confirm the reliability of the initial screening data, nematode reproductive phenotype data obtained from the high‐throughput screening platform were combined with the least absolute shrinkage and selection operator (LASSO) regression algorithm and metabolomics data of single‐gene knockout *E. coli* strains obtained from public databases. The calculation results of the Lasso regression algorithm show that a total of 68 metabolites obtained nonzero coefficients, indicating a significant correlation between 68 metabolites and nematode reproductive phenotype data (Figure 1C,D). Afterwards, the extracted 68 metabolite data were used as a new data matrix, and the consistency clustering method was used to cluster the data matrix. The area under the CDF curve was selected to choose cluster number 6 as the optimal cluster number (Figure 1E). Twenty‐nine metabolites were identified as functionally enriched in reproductive‐related pathways, including pantothenic acid biosynthesis, pentose phosphate pathway, pyruvate metabolism and glycolysis (Figure 1F).

### 2.2 *E. coli* Mutants Significantly Improve Fertility in CTX‐Induced Reproductive Disorder in *C. elegans*

To investigate the role of *E. coli* mutants that increased nematode fertility in the context of CTX‐induced male reproductive toxicity, a CTX‐induced reproductive disorder (CTXD) *C. elegans* model was constructed. First, N2 nematodes were treated with three gradients of CTX, namely 500 μg/mL, 1 mg/mL and 2 mg/mL. The total number and daily number of offspring produced by nematodes were counted. Compared with the blank group, the offspring number was significantly reduced in the CTX‐treated groups. Offspring numbers decreased with increasing CTX concentrations from 500 μg/mL to 2 mg/mL. A 1 mg/mL CTX concentration reduced offspring numbers by 50% (Figure 1G,H), making it the optimal concentration for constructing the CTXD *C. elegans* models. Second, the 46 mutant strains that significantly enhanced *C. elegans* fertility were fed to CTXD nematodes. The daily offspring numbers were counted. Twenty‐six mutants increased offspring numbers by 50% in CTXD nematodes, compared with the control group. These strains were identified as *E. coli* mutants capable of improving fertility in CTXD nematodes and were ranked according to their reproductive ratios (Figure 1I,J).

### 2.3 Engineering Probiotics Ecn Δ*pal* Significantly Ameliorates CTX‐Induced Male Reproductive Toxicity

**FIGURE 2:**
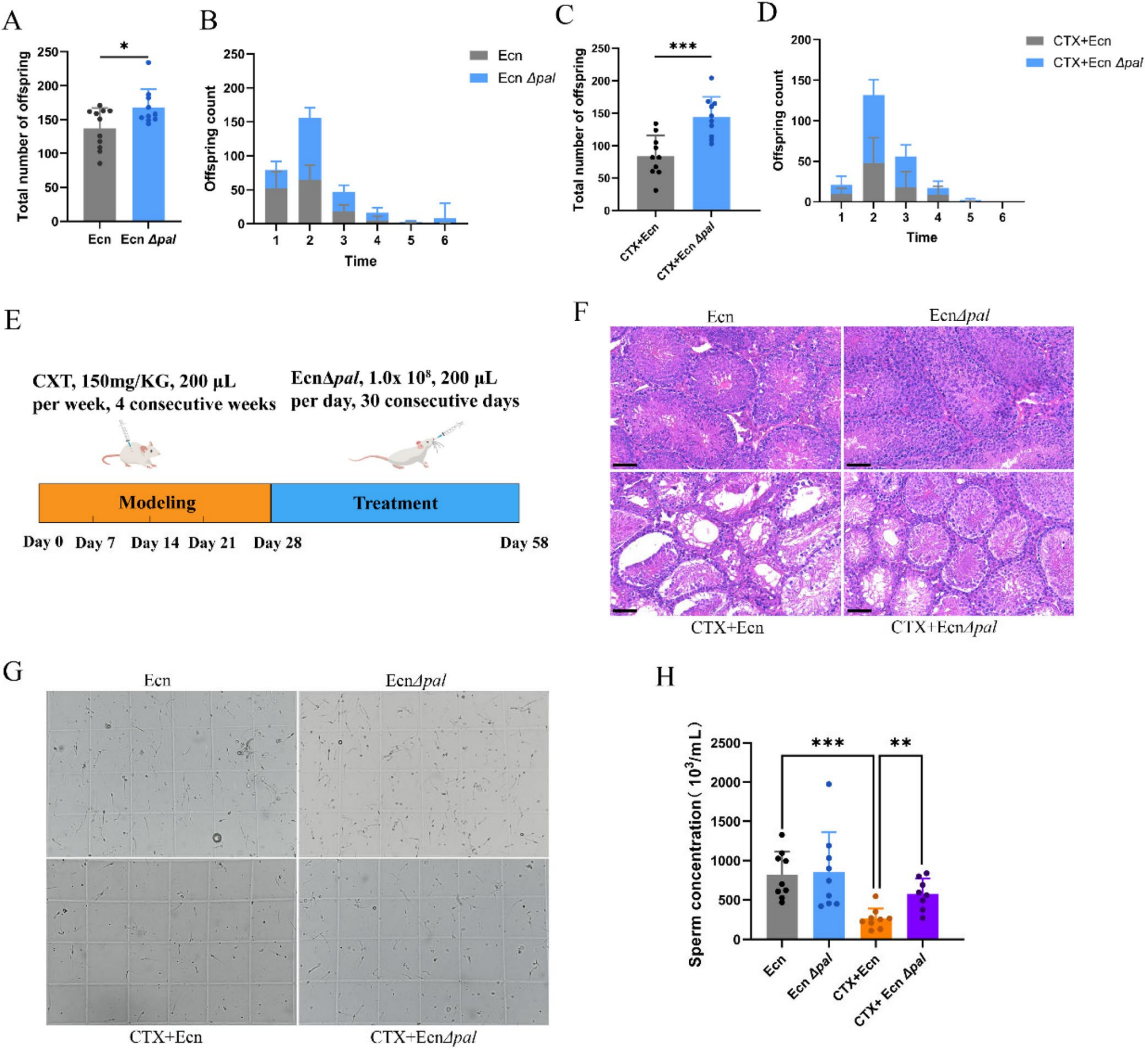
Engineering probiotic Ecn Δ*pal* significantly mitigates CTX‐induced male reproductive toxicity. (A) Total offspring number produced by N2 nematodes after being fed on Ecn and Ecn Δ*pal*. (B) Daily offspring number produced by N2 nematodes after being fed on Ecn and Ecn Δ*pal*. (C) Total offspring number produced by CTXD N2 nematode after being fed on Ecn and Ecn Δ*pal*. (D) Daily offspring number produced by N2 CTXD nematode after being fed on Ecn and Ecn Δ*pal*. (E) Schematic diagram depicting CTXD mouse model construction and Ecn Δ*pal* treatment. (F) Ecn Δ*pa*l mitigates tissue disruption in testicular pathology observed in CTXD mice (HE staining, Scale bar = 50 μm). (G) Representative images of sperm morphology capture. (H) Quantitative analysis of sperm count. Error bars show mean ± SEM (nematodes: *n* = 10). ice: n=6 per group. *p* values are from unpaired t‐tests. *p < 0.05; ***p < 0.001. CTX, cyclophosphamide; CTXD, CTX‐induced reproductive disorder; Ecn, *Escherichia coli* Nissle 1917; Ecn Δ*pal*, *E. coli* Nissle 1917 Δ*pal*; H&E, haematoxylin and eosin and SEM, standard error of the mean.

We explored whether these single‐gene knockouts exert similar effects on Ecn for the development of engineered probiotics. Five mutant strains (leading five) showing the most significantly enhanced CTX‐induced fertility in impaired *C. elegans* were engineered as probiotics using Clustered Regularly Interspaced Short Palindromic Repeats‐Cas9 gene editing technology. These mutant strains were fed to *C. elegans* and CTXD *C. elegans* . The offspring number produced by nematodes daily and their total offspring were recorded. The results showed that only the top two, namely Ecn Δ*pal* and Ecn Δ*yghZ*, could improve the reproduction of CTXD mice, and the knockout strain effect of Ecn Δ*pal* was significantly better than that of Ecn Δ*yghZ* (Figures 2 and S1). Nematodes fed with Ecn ∆*pal* produced 22.46% more offspring than those fed with Ecn (*p* < 0.05) (Figure 2A). Additionally, the daily offspring number was higher in nematodes fed with Ecn ∆*pal* than in those fed with Ecn (Figure 2B). CTXD nematodes fed with Ecn ∆*pal* increased total offspring by 70.98% (*p* < 0.001) (Figure 2C), and their daily offspring number was higher than that in the control group (Figure 2D). A CTXD male mouse model was constructed by intraperitoneally injecting microbiota‐depleted male mice with 159 mg/kg CTX weekly for four consecutive weeks. Subsequently, the mice were administered Ecn ∆*pal* (1.0 × 10^8^, 200 μL) orally for 30 consecutive days (Figure 2E). A blank control group (fed with Ecn) and a positive control group (fed with Ecn ∆*pal*) were set up for comparison. On day 59, mice were humanely killed, and their sperm quality was analysed. Mice in the Ecn and Ecn ∆*pal* groups demonstrated normal testicular tissue development, with intact seminiferous epithelium, normal sperm production and regular basement membrane. The sperm count in the Ecn ∆*pal* group was insignificantly higher than that in the Ecn group. Compared with the Ecn group, the CTX + Ecn group demonstrated a significant decrease in sperm count and disrupted testicular tissue structure, including severe atrophy of the seminiferous tubules, and spermatogonia shedding. However, after treatment with Ecn ∆*pal*, the sperm count in CTXD male mice significantly increased compared with the CTX + Ecn group. The testicular tissue partially recovered its morphology (Figure 2F–H). Moreover, the knockout strain effect of Ecn Δ*pal* is significantly better than that of Ecn Δ*yghZ* (Figure S1F,J).

### 2.4 Purine Metabolism Is a Key Pathway to Mitigate Spermatogenesis in CTXD Male Mice

**FIGURE 3:**
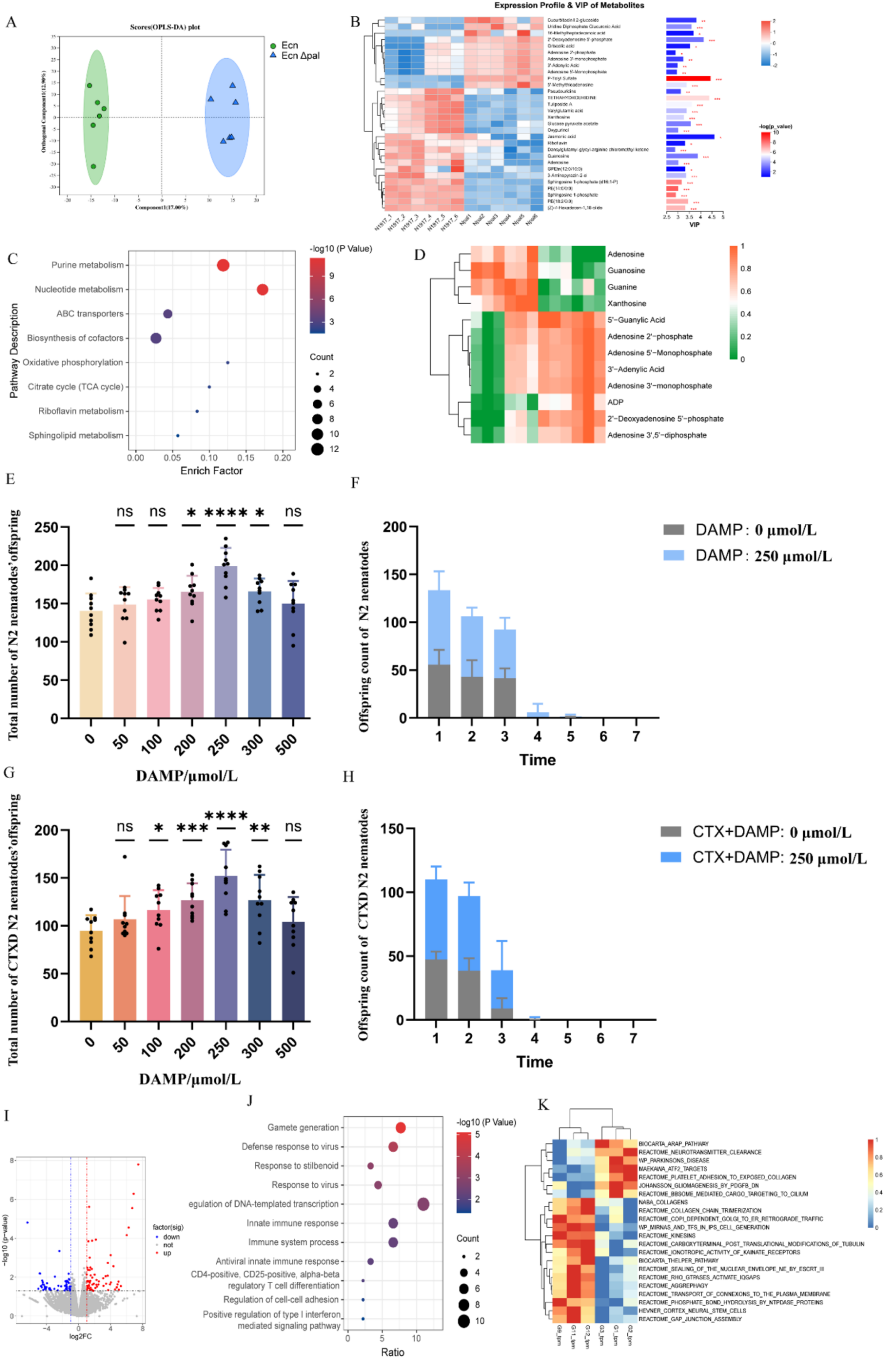
Metabolome analysis of Ecn and Ecn Δ*pal* strains and transcriptome analysis of the testes of CTXD mice fed with Ecn and Ecn Δ*pal* strains. (A) OPLS‐DA of samples in Ecn and Ecn Δ*pal* strains groups. Green circles represent samples from the Ecn group, whereas blue triangles represent samples from the Ecn Δ*pal* group. (B) Heatmap showing differential metabolites between Ecn and Ecn Δ*pal*. (C) KEGG enrichment analysis of differential metabolites between Ecn Δ*pal* and Ecn strains. (D) Heatmap showing differential metabolites within the purine metabolism pathway in Ecn and Ecn Δpal strains. (E) Total offspring number produced by N2 nematodes after being fed on different concentrations of DAMP. (F) Daily offspring number produced by N2 nematodes after being fed on DAMP at a concentration of 250 μmol/L. (G) Total offspring number produced by CTXD N2 nematode after being fed on different concentrations of DAMP. (H) Daily offspring number produced by CTXD N2 nematodes after being fed on DAMP at a concentration of 250 μmol/L. (I) Volcano plot showing differential gene expression (upregulated, red; downregulated, blue) between the testes of CTXD mice fed with Ecn and Ecn Δ*pal*. (J) GO enrichment analysis of differential genes between the testes from CTXD mice fed with Ecn and Ecn Δ*pal*. (K) ssGSEA based on gene expression data from CTXD mice. OPLS‐DA, orthogonal partial least squares discriminant analysis; Ecn, *Escherichia coli* Nissle 1917; Ecn Δ*pal*, *E. coli* Nissle 1917 Δpal; CTXD, cyclophosphamide‐induced reproductive disorder; DAMP, metabolite 2′‐Deoxyadenosine 5′–phosphate; KEGG, Kyoto Encyclopedia of Genes and Genomes and GO, Gene Ontology.

To clarify the metabolic differences between Ecn Δpal and Ecn strains, metabolomics sequencing was conducted on both strains. First, orthogonal partial least squares discriminant analysis was conducted to establish a relationship model between metabolite levels and differences among samples. Ecn Δpal and Ecn exhibited significant differences in metabolic states (Figure 3A). Compared with Ecn, Ecn Δpal exhibited 118 significantly different metabolites, including 39 upregulated and 79 downregulated metabolites (Figure 3B and Figure S2). Kyoto Encyclopedia of Genes and Genomes (KEGG) pathway enrichment analysis demonstrated that these differential metabolites were significantly enriched in pathways such as purine metabolism, nucleotide metabolism and ABC transporters (Figure 3C). Upregulated metabolites in the pathway included ADP and 3′‐adenolic acid, among others (Figure 3D). To determine which component of the mutant improves host reproduction, we added metabolites significantly upregulated by the Ecn Δ*pal* mutant to the nematode culture medium, rather than the Ecn Δ*pal* mutant. The metabolite 2′‐deoxyadenosine 5′‐phosphate (DAMP, Figure 3B) was found to significantly enhance the reproductive ability of N2 nematodes and CTXD N2 nematodes, especially at a concentration of 250 μmol/L, indicating that an appropriate dose of DAMP is beneficial for host reproduction (Figure 3E–H).

To investigate the mechanism by which Ecn ∆*pal* improves spermatogenesis in CTXD male mice, transcriptome sequencing was conducted on the CTXD testes of mice orally administered Ecn ∆*pal* and Ecn. Differential expression analysis identified 121 significantly different genes between the groups, including 62 upregulated and 59 downregulated genes (Figure 3I). Gene type annotation identified 50 protein‐coding genes (41.32%) and 33 lncRNAs (27.27%) (Figure S3). Gene Ontology enrichment analysis suggested that differentially expressed genes were significantly enriched in biological processes associated with gamete generation (Figure 3J). Based on gene expression data, single sample gene set enrichment analysis identified 22 pathways with significant differences between the Ecn ∆*pal* and Ecn groups, including BIOCARTATA_HELPER‐PATHWAY and REACTONOTROPIC‐ACTIVITY OF KAINATE RECEPTORS (Figure 3K).

## 3 Discussion

Research on the regulation of male reproduction by the human gut microbiota has increased considerably. Changes in the composition and functionality of gut microbiota directly affect human health and are central to the onset of numerous diseases (Hou et al. 2022). *C. elegans* fed with different laboratory bacterial strains exhibit remarkable physiological differences in growth rate, life span and reproduction (Brooks et al. 2009; MacNeil et al. 2013; Zhang et al. 2017). Genetic variations in gut microbiota can also significantly affect the physiological functions of the host. For instance, Wu et al. showed that a specific *E. coli* structural variation (SV)—scrK deletion—alters fructose metabolism and exacerbates colitis in mice on a high‐fructose diet (Wu et al. 2025). Our study highlights the importance of microbial genetic diversity in modulating host reproduction. By utilising *C. elegans* as a model organism, we demonstrated that the deletion of specific bacterial genes can affect host reproduction. Through this approach, we identified 46 bacterial mutants that improved nematode fertility. This research is a substantial and expensive endeavour; however, it enables the rapid and accurate screening of strains significantly affecting nematode fertility through a high‐throughput screening platform and nematode number identification software. This lays the foundation for further research. Interestingly, among these 46 bacterial mutants, 26 strains enhanced fertility in *C. elegans* with CTX‐induced reproductive disorders.

CTX—a common chemotherapy drug—effectively treats cancer but can produce some adverse effects, such as male reproductive toxicity. These adverse effects comprise azoospermia, oligospermia, atrophy of testicular seminiferous tubules and decreased serum testosterone levels (Abarikwu et al. 2012; Gerres et al. 1998; Pectasides et al. 2009), which severely threatens fertility in men of reproductive age. No effective clinical methods exist to prevent these adverse effects. Some herbal extracts mitigate CTX‐induced testicular damage; nonetheless, more effective ‘drugs’ warrant investigation.

Probiotics are one of the most widely used alternatives for disease prevention and treatment. In contrast, for faecal transplantation, probiotics can be standardised. However, the poor effectiveness of a probiotic hinders its widespread application. This warrants improving their formulation and functionality (Abdollahi‐Roodsaz et al. 2016). Genetic engineering facilitates the creation of next‐generation probiotics. Our study suggests that microbial genetic variations can enhance host reproductive capacity and mitigate CTX‐induced male reproductive toxicity, contributing to the development of reproductive probiotics. In this study, we engineered probiotics and observed that Ecn Δ*pal* significantly improved spermatogenesis in CTXD male mice. Therefore, specific genetic variations may enhance the functionality of probiotics, despite the lack of reports on probiotics improving CTX‐induced male reproductive toxicity.

To investigate the mechanisms by which Ecn Δ*pal* improves spermatogenesis in CTXD male mice, we conducted a metabolomic analysis of Ecn ∆*pal* strains and a transcriptomic analysis of the CTXD testes of male mice colonised with Ecn ∆*pal*. Differential metabolites were significantly enriched in purine metabolism, nucleotide metabolism and ABC transporters pathways, whereas differentially expressed genes were significantly enriched in gamete generation processes. In addition, the experimental results suggest that an appropriate dose of DAMP, a metabolite significantly upregulated by Ecn ∆*pal*, may ameliorate host reproduction. DAMP is an intermediate in the synthesis of deoxyadenylic acid in the purine nucleotide synthesis pathway and a key nucleotide for DNA synthesis. It is involved in cellular processes such as DNA replication, repair and gene expression regulation (Duarte et al. 1999; Narumi et al. 2019). During the reproductive process, cellular division and embryonic development cannot proceed without the normal synthesis and repair of DNA, and its role runs through the entire reproductive and embryonic development processes (García‐Rodríguez et al. 2019; Jaroudi et al. 2009; Mundt et al. 2022). These are consistent with our findings that purine nucleotide DAMP may be the key component through which Ecn ∆*pal* improves host reproduction. Notably, using phenotypic and metabolomics data from 3467 mutant strains, we applied the LASSO regression method to identify reproduction‐related metabolites, which were also significantly enriched in the purine metabolism pathway. In summary, Ecn ∆*pal* may upregulate purine nucleotides, such as DAMP, through the purine metabolic pathway, thereby improving spermatogenesis in CTXD mice.

## 4 Materials and Methods

### 4.1 *E. coli* ‐ *C. elegans* High Throughput Screening Platform

The *E. coli* Keio Knockout Collection was used as the experimental strain, and the parental strain BW25113 of the library was used as the control group. After the synchronisation of N2 nematodes, the single‐gene knockout strains and the control bacteria were fed to them. There were 10 parallel groups for each type of mutant strain, with one nematode in each group. The number of nematode offspring was recorded by Worm Studio, a nematode number counting software developed by the research group. Finally, a single gene knockout *E. coli* was preliminarily screened to improve the fertility of N2 nematodes. The above forms an *E. coli–C. elegans* high throughput screening platform.

### 4.2 *E. coli* Single‐Gene Knockout Ecn Mutant Constructed by CRISPR‐Cas9 Technology

Small guide RNA (sgRNA) sequences were designed on http://crispr.dfci.harvard.edu/SSC/ and synthesised by Sangon Biotech (Shanghai) Co. Ltd.

The donor DNA consists of approximately 500 bp gene fragments upstream and downstream of the target gene. Using the Ecn genome as a template, primers (*pal*‐19‐F/pal—up‐R, pal‐down‐F/*pal*‐19‐R) were designed to amplify fragments containing enzyme cleavage sites, homologous sequences of PUC19 plasmid and upstream and downstream fragments of the target gene. The upstream and downstream fragments were seamlessly connected to the pUC19 plasmid, that is, UD‐pUC19 plasmid. Design primers (*pal*‐up‐F/*pal*‐down‐R), using the UD‐pUC19 plasmid sequenced correctly as a template to amplify ‘repairing homologous arms’, that is, donor DNA.

Electrotransformation of Cas9 containing plasmids into Ecn, preparation of Cas9 Ecn competent cells by CaCl2 method. Finally, transduction of donor DNA and sgRNA plasmids into Cas9 Ecn competent cells by electroporation. The primers and sgRNA sequence for constructing Ecn Δ *pal* in this study are as follows:

**Table:** 

sgRNA	TTCTGACTTCGCTCAAATGC
*Pal*‐up‐F	AGGATGCGGATGTCAGCAGCGAC
*Pal*‐up‐R	CTGCTCATGCAATTCTCTTCAATGATTCCTTTACTATTCA
*pal*‐down‐F	AGTAAAGGAATCATTGAAGAGAATTGCATGAGCAGTAACT
*pal*‐down‐R	CTGGTTGATTGCGCCGCTGCACC
*pal*‐19‐F	TGTAAAACGACGGCCAGTAGGATGCGGATGTCAGCAGCGAC
*pal*‐19‐R	CTATGACCATGATTACGCCTGGTTGATTGCGCCGCTGCACC

### 4.3 Construction of CTX‐Induced Reproductive Disorder N2 *C. elegans* Model

Synchronised L1 N2 *C. elegans* were diluted to 20–30 cells/10 μL and were collected with S‐complete medium buffer to culture in 96‐well plates (365 μL/well). One hundred and sixty‐five microliters of S‐complete medium buffer, 20 μL of 10 mg/mL CTX, 10 μL of L1 nematodes and 5 μL of OP50 were added to 96‐well plates and incubated in a biological incubator at 20°C for 72 h (up to adult DAY1), then fed to Ecn or Ecn mutants. A concentration of 1 mg/mL of CTX was used to construct a CTX‐induced reproductive disorder N2 *C. elegans* model, abbreviated as CTXD *C. elegans* . This concentration can reduce the fertility of nematodes by 50%, compared to the blank control (CTX concentration of 0 mg/mL).

### 4.4 Construction of CTX‐Induced Reproductive Disorder Male Mice Model and Treatment

6‐ to 8‐week‐old C57BL/6J male mice were treated with a cocktail of broad‐spectrum antibiotics (Solarbio) in their drinking water for 1 week to prepare the microbiota‐depleted male mice (Li et al. 2017). These antibiotics include Ampicillin (1 mg/mL), Neomycin sulphate (1 mg/mL), Metronidazole (1 mg/mL) and Vancomycin (0.5 mg/mL). After antibiotic treatment, inject 150 mg/kg CTX into the microbiota‐depleted male mice intraperitoneally according to body weight once a week for four consecutive weeks. Then, administer mutant Ecn or Ecn mutants suspension (1 × 10^8^ CFU in 200 μl PBS) orally to CTX‐induced reproductive disorder male mice (abbreviated as CTXD mice) daily for 30 consecutive days. Mice have been approved by the Medical Ethics Committee of Xuzhou Medical University, with animal ethics number 202302 T006.

### 4.5 Sperm Counting

Transfer the bilateral epididymis of each mouse to 2 mL of physiological saline and homogenise for 30 s. Place in a 37°C constant temperature incubator and incubate for 30 min to fully release the sperm into the physiological saline solution. Dilute the sperm suspension and place it on a sperm counting plate. Cover it with a glass slide and observe and count it using an optical microscope.

### 4.6 Metabolomic Analysis

Untargeted metabolomics analysis was performed at Majorbio, China, on a platform consisting of an independent ultra‐high performance liquid chromatography–tandem mass spectrometry (UPLC‐MS/MS) instrument. In short, each group collected testicular tissue for metabolite extraction. The mass spectrometric data were collected using a Thermo UHPLC‐Q Exactive HF‐X Mass Spectrometer equipped with an electrospray ionisation (ESI) source operating in positive mode and negative mode. The raw data from the mass spectrometry instrument were imported into the commercial software Progenesis QI (version 2.2) for peak extraction, obtaining information such as mass‐to‐charge ratio, retention time and ion area related to metabolites. Using the multivariate analysis OPLS‐DA model, combined with *p* values by Student's t test, we screened differentially expressed metabolites with VIP > 1 and *p* < 0.05. Pathway enrichment analysis of differential metabolites was completed using MetaboAnalyst 6.0 (PMID: 38587201).

### 4.7 RNA Extraction and Whole Transcriptome RNA Resequencing

RNA was extracted from mice testicular tissue using the TRIzol Reagent. Then RNA quality was determined by 5300 Bioanalyser (Agilent) and quantified using the ND‐2000. After quality control through electrophoresis, the extracted RNA underwent purification, rRNA removal, fragmentation, cDNA synthesis, terminal repair process and PCR amplification. The concentration of the constructed libraries was measured using a Qubit4.0, and the libraries were sequenced on an Illumina NovaSeq Xplus sequencer. Applying fastp (https://github.com/OpenGene/fastp) to filter Reads to obtain clean Reads. The split mapping algorithm of Hisat2 was used to perform Genome mapping on preprocessed reads (PMID: 31375807). Count number of Fragments for each gene after Hisat2 alignment using RSEM (PMID: 21816040). Genes with absolute value of logFC > 1 and *p* < 0.05 were considered as significantly differentially expressed genes using DESeq2 (PMID: 25516281). GO functional enrichment analysis of differentially expressed genes was used DAVID web server (PMID: 35325185).

### 4.8 Screening of Differential Pathways Based on RNA‐Seq Data

Single‐sample gene set enrichment analysis (ssGSEA) is a method used to evaluate the activity of a certain gene set or pathway for a sample. We obtained the M2 dataset of mice (2710 gene sets) from the Molecular Signature Database. Based on RNA‐seq data from mice testes, ssGSEA scores were calculated for 2710 pathways using the GSVA R package (PMID: 23323831). Differential analysis was performed on 2710 pathways by Student's t test. The ssGSEA score was transformed using 0–1 normalisation for comparison in the heatmap.

### 4.9 Statistical Analysis

Data are shown as “Mean ± SEM” for each experiment. The statistical analysis was performed using GraphPad Prism 8.0 software. The significance of differences between groups was analysed using the unpaired *t* test, and if the variance is not homogeneous, a nonparametric test method is used. Values of *p* < 0.05 were considered significant. The number of samples is indicated in the description of each experiment.

## Author Contributions

Z.Z., Y.L. and X.D. designed the experiments, conducted data analysis and drafted the manuscript. X.D., Y.W., T.Z. and R.G. performed the majority of the experiments. W.C., Y.R., Y.L., C.W., A.T., X.D., Y.L. and Z.Z. discussed the data and its interpretation. D.Z. carried out data analysis. Y.L. provided technical support throughout the study.

## Conflicts of Interest

The authors declare no conflicts of interest.

## Supporting information


**Figure S1:** The knockout strain effect of Ecn Δpal was significantly better than that of Ecn Δ*yghZ*. (A) Total offspring number produced by N2 nematodes after feeding on Ecn and Ecn Δ*yghZ*. (B) Daily offspring number produced by N2 nematodes after feeding on Ecn and Ecn Δ*yghZ*. (C) Total offspring number produced by CTXD N2 nematode after feeding on Ecn and Ecn Δ*yghZ*. (D) Daily offspring number produced by N2 CTXD nematode after feeding on Ecn and Ecn Δ*yghZ*. (E) Relative total offspring number produced by N2 nematodes after feeding on Ecn, Ecn Δ*pal* and Ecn Δ*yghZ*. (F) Relative total offspring number produced by CTXD N2 nematodes after feeding on Ecn, Ecn Δ*pal* and Ecn Δ*yghZ*. (G) Ecn Δ*yghZ* mitigates tissue disruption in testicular pathology observed in CTXD mice (HE staining, Scale bar = 50 μm). (H) Representative images of sperm morphology capture. (I) Quantitative analysis of sperm count. (J) Relative quantitative analysis of sperm count. Error bars show mean ± SEM (*n* = 10). *n* = 6 mice per group. *p* values are from unpaired t‐tests. *p < 0.05; ***p < 0.001. CTX, cyclophosphamide; CTXD, CTX‐induced reproductive disorder; Ecn, *Escherichia coli* Nissle 1917; Ecn Δ*yghZ*, *E. coli* Nissle 1917 Δ*pal*; H&E, haematoxylin and eosin and SEM, standard error of the mean.
**Figure S2:** Heatmap illustrating differential metabolites between Ecn and Ecn Δ*pal*.
Ecn, *Escherichia coli* Nissle 1917; Ecn Δ*pal*, *E. coli* Nissle 1917 Δpal.
**Figure S3:** Annotation of gene types for 121 differentially expressed genes between the testes of mice fed with Ecn and Ecn Δ*pal*.
Ecn, *Escherichia coli* Nissle 1917; Ecn Δ*pal*, *E. coli* Nissle 1917 Δpal.

## Data Availability

The data that support the findings of this study are available from the corresponding author upon reasonable request.
