# Supplementary material for: Gut Microbial Genetic Variation Regulates Host Reproduction
Source: Microb Biotechnol. 2025 Oct 10;18(10):e70248. doi: 10.1111/1751-7915.70248 (PMC12511951; doi:10.1111/1751-7915.70248)
Supplement: Supplementary file 1 — Figure S1: The knockout strain effect of Ecn Δpal was significantly better than that of Ecn ΔyghZ. (A) Total offspring number produced by N2 nematodes after feeding on Ecn and Ecn ΔyghZ. (B) Daily offspring number produced by N2 nematodes after feeding on Ecn and Ecn ΔyghZ. (C) Total offspring number produced by CTXD N2 nematode after feeding on Ecn and Ecn ΔyghZ. (D) Daily offspring number produced by N2 CTXD nematode after feeding on Ecn and Ecn ΔyghZ. (E) Relative total offspring number produced by N2 nematodes after feeding on Ecn, Ecn Δpal and Ecn ΔyghZ. (F) Relative total offspring number produced by CTXD N2 nematodes after feeding on Ecn, Ecn Δpal and Ecn ΔyghZ. (G) Ecn ΔyghZ mitigates tissue disruption in testicular pathology observed in CTXD mice (HE staining, Scale bar = 50 μm). (H) Representative images of sperm morphology capture. (I) Quantitative analysis of sperm count. (J) Relative quantitative analysis of sperm count. Error bars show mean ± SEM (n = 10). n = 6 mice per group. p values are from unpaired t‐tests. *p < 0.05; ***p < 0.001. CTX, cyclophosphamide; CTXD, CTX‐induced reproductive disorder; Ecn, Escherichia coli Nissle 1917; Ecn ΔyghZ, E. coli Nissle 1917 Δpal; H&E, haematoxylin and eosin and SEM, standard error of the mean. Figure S2: Heatmap illustrating differential metabolites between Ecn and Ecn Δpal. Ecn, Escherichia coli Nissle 1917; Ecn Δpal, E. coli Nissle 1917 Δpal. Figure S3: Annotation of gene types for 121 differentially expressed genes between the testes of mice fed with Ecn and Ecn Δpal. Ecn, Escherichia coli Nissle 1917; Ecn Δpal, E. coli Nissle 1917 Δpal. [file MBT2-18-e70248-s001.docx]

**Supplementary**

**
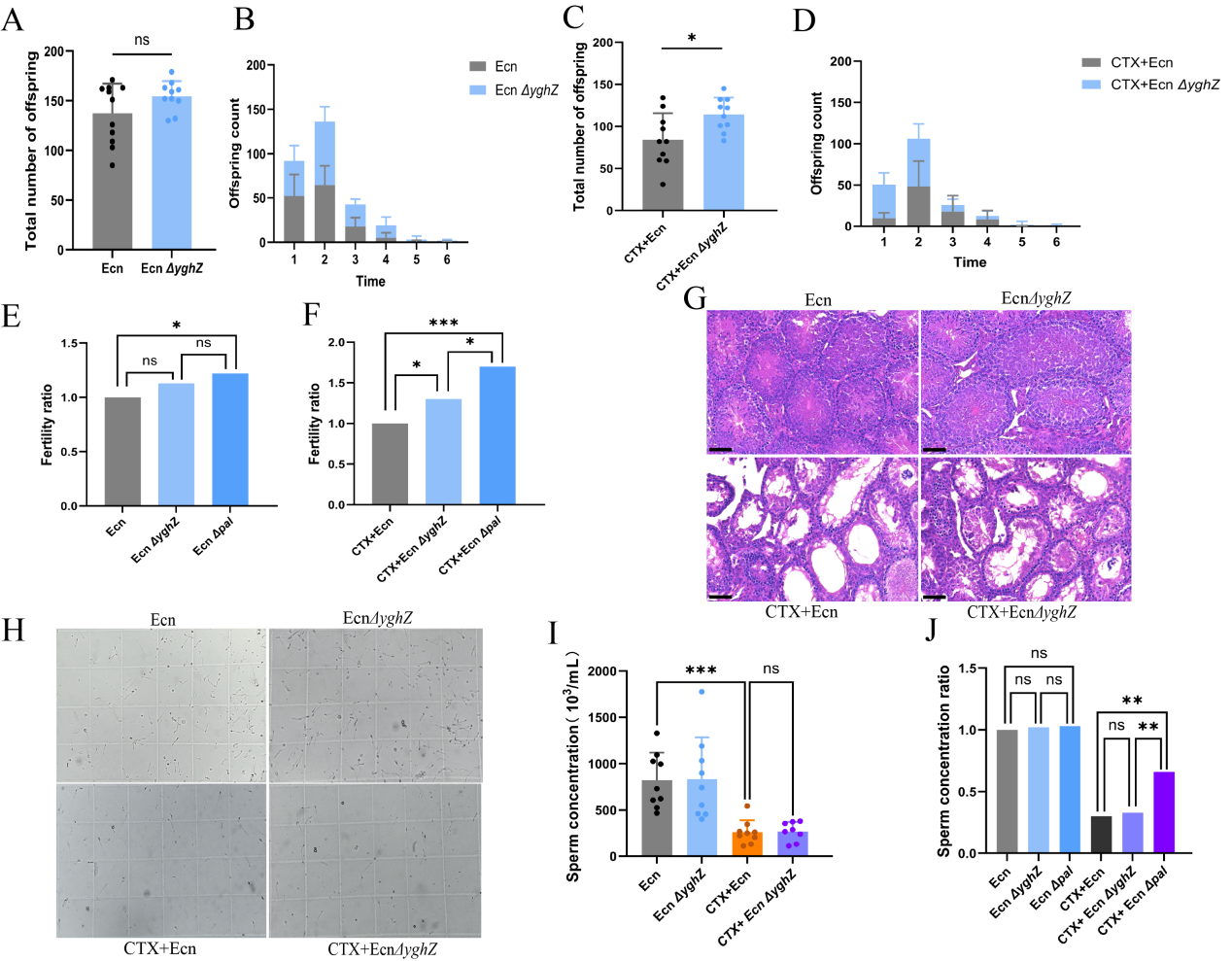
**

**Supplementary Figure 1. The knockout strain effect of Ecn Δpal was significantly better than that of Ecn Δ*yghZ*.** (A) Total offspring number produced by N2 nematodes after feeding on Ecn and Ecn Δ*yghZ*. (B) Daily offspring number produced by N2 nematodes after feeding on Ecn and Ecn Δ*yghZ*. (C) Total offspring number produced by CTXD N2 nematode after feeding on Ecn and Ecn Δ*yghZ*. (D) Daily offspring number produced by N2 CTXD nematode after feeding on Ecn and Ecn Δ*yghZ*. (E) Relative total offspring number produced by N2 nematodes after feeding on Ecn, Ecn Δ*pal* and Ecn Δ*yghZ*. (F) Relative total offspring number produced by CTXD N2 nematodes after feeding on Ecn, Ecn Δ*pal* and Ecn Δ*yghZ*. (G) Ecn Δ*yghZ* mitigates tissue disruption in testicular pathology observed in CTXD mice (HE staining, Scale bar=50 μm). (H) Representative images of sperm morphology capture. (I) Quantitative analysis of sperm count. (J) Relative quantitative analysis of sperm count. Error bars show mean ± SEM (n=10). n = 6 mice per group. P-values are from unpaired t-tests. * P<0.05; ***P<0.001. CTX, cyclophosphamide; CTXD, CTX-induced reproductive disorder; Ecn, Escherichia coli Nissle 1917; Ecn Δ*yghZ*, E. coli Nissle 1917 Δ*pal*; H&E, hematoxylin and eosin; and SEM, standard error of the mean

**
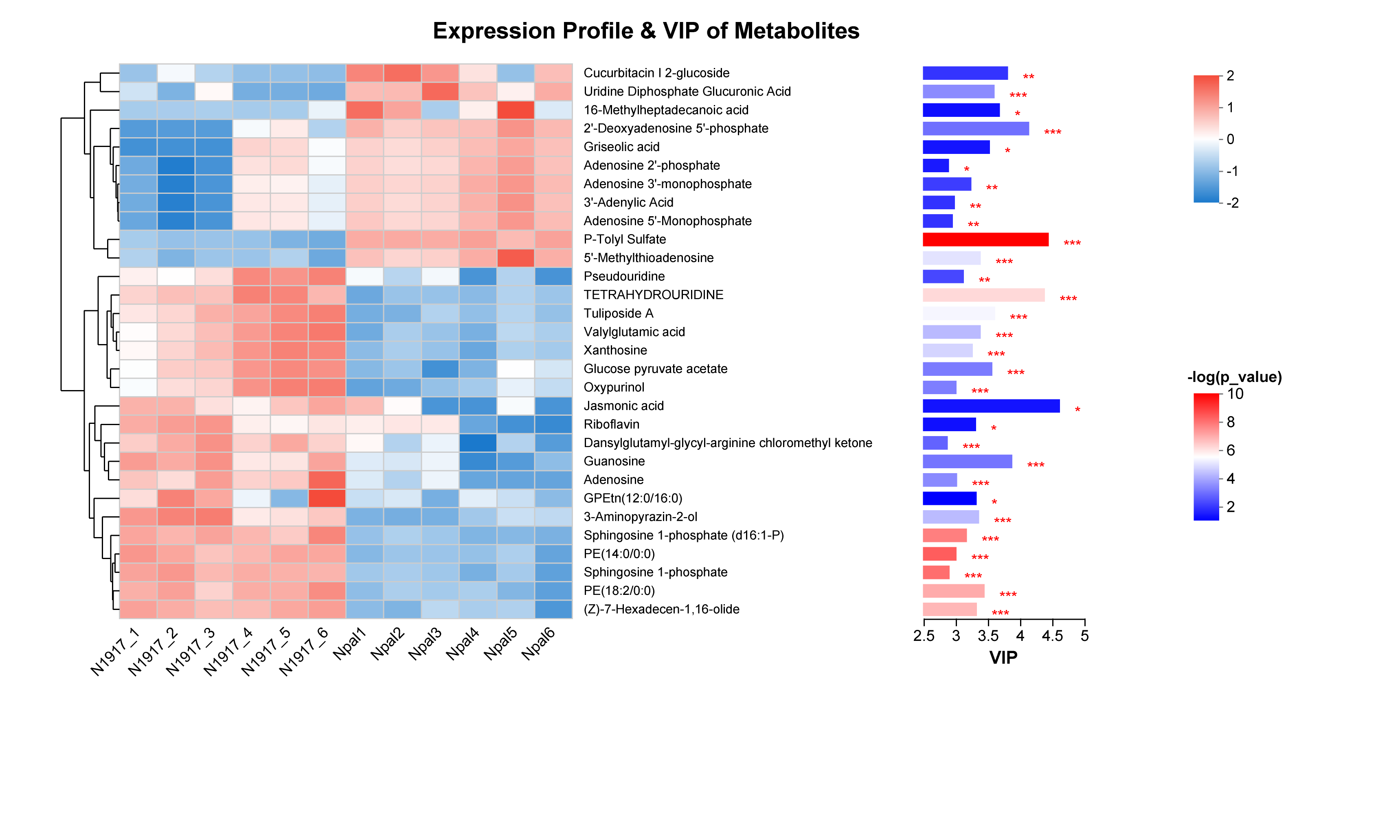
**

**Supplementary Figure 2. Heatmap illustrating differential metabolites between Ecn and Ecn Δ*pal***

Ecn, *Escherichia coli* Nissle 1917; Ecn Δ*pal*, *E. coli* Nissle 1917 Δpal

**
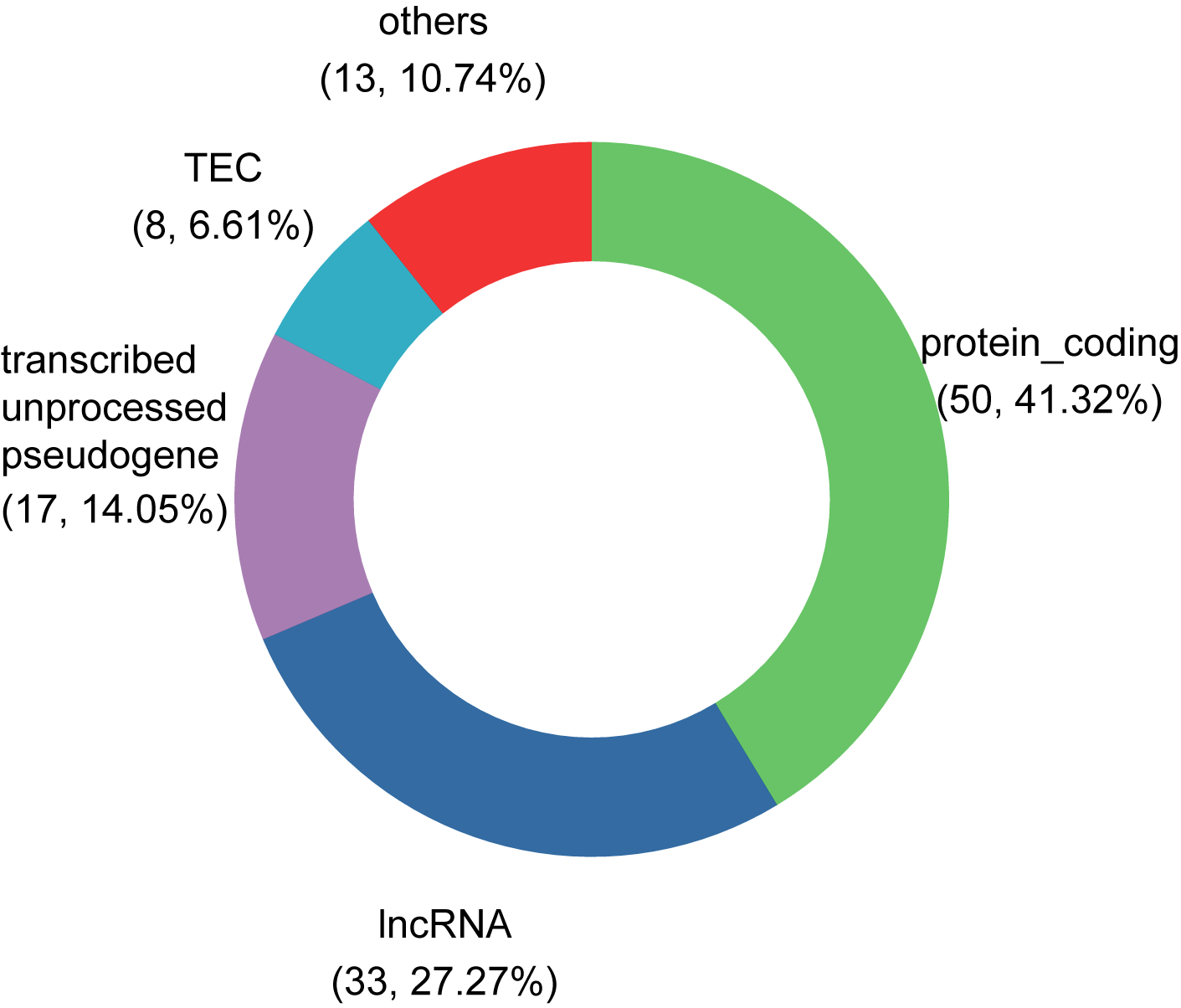
**

**Supplementary Figure 3. Annotation of gene types for 121 differentially expressed genes between the testes of mice fed with Ecn and Ecn Δ*pal***

Ecn, *Escherichia coli* Nissle 1917; Ecn Δ*pal*, *E. coli* Nissle 1917 Δpal
